# Supplementary material for: 3D ocean assessments reveal that fisheries reach deep but marine protection remains shallow
Source: Nat Commun. 2024 May 21;15:4027. doi: 10.1038/s41467-024-47975-1 (PMC11109251; doi:10.1038/s41467-024-47975-1)
Supplement: Supplementary file 1 — Supplementary Information [file 41467_2024_47975_MOESM1_ESM.pdf]

3D ocean assessments reveal that fisheries reach deep but marine protection remains shallow

Juliette Jacquemont[1,2*], Charles Loiseau[2], Luke Tornabene[1], Joachim Claudet[2*]

Corresponding author: juliette.jacquemont.fr@gmail.com

The PDF file includes:

Supplementary Figures 1 to 5
Supplementary Tables 1 to 4

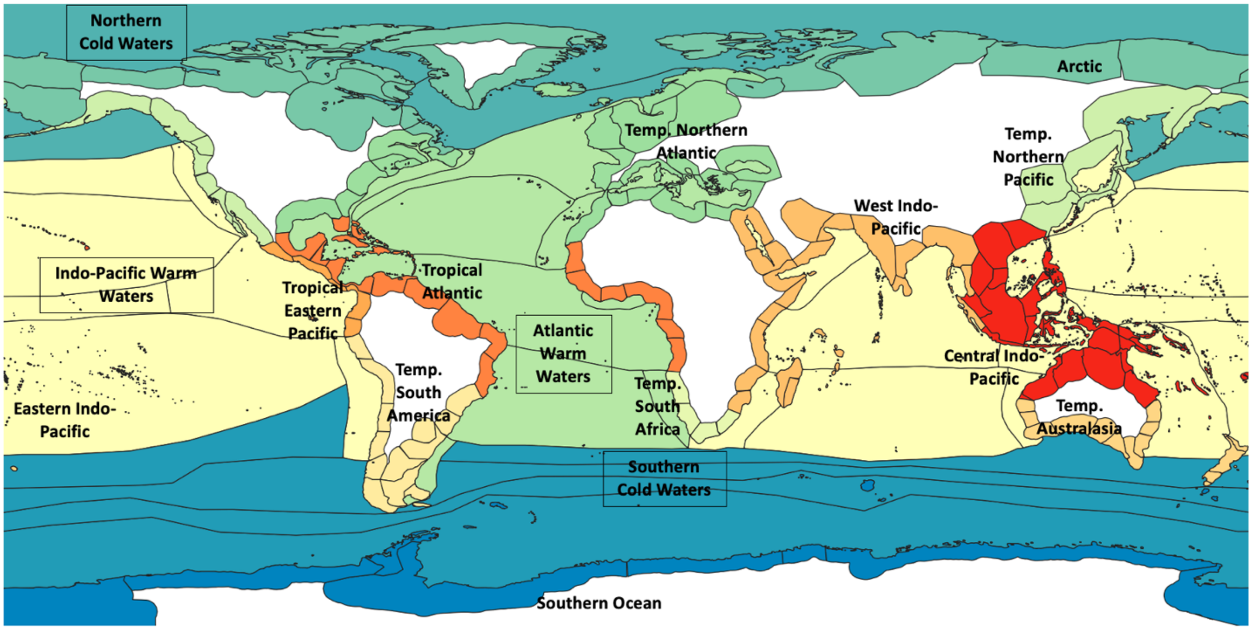

Fig. S1. Location of 2D marine ecoregions. Off-shore ecoregions are indicated by square outlines. Each ecoregion is depicted in a different color. Black lines represent the limits of pelagic provinces, a subdivision of off-shore realms defined in Spalding et al., (2012) but not considered in our analysis.

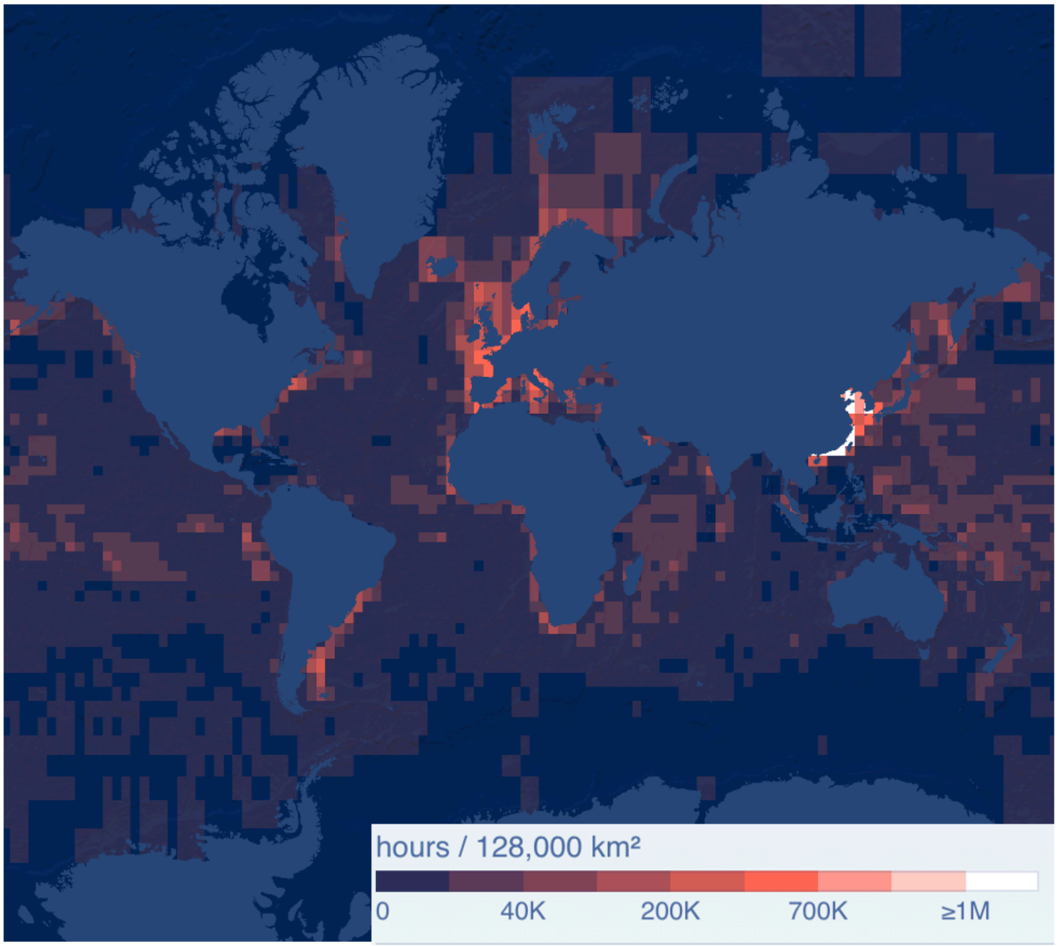

Fig. S2. Total fishing hours per unit area in 2019. Data and map display from the Global Fishing Watch online interface.

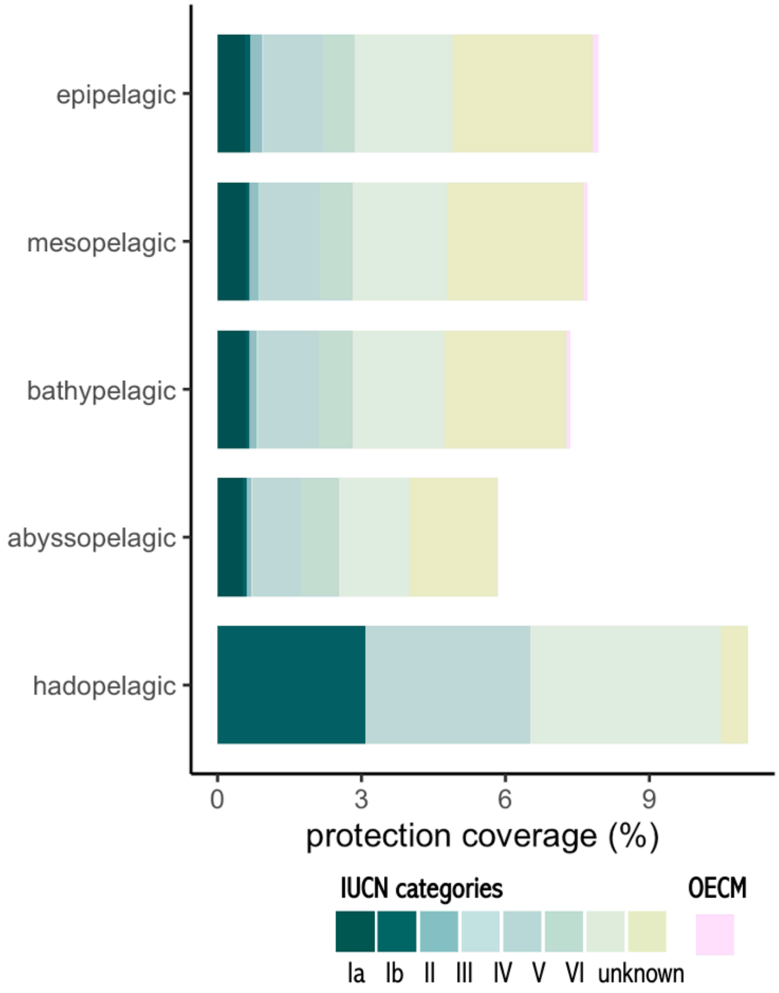

Fig. S3. Distribution of protection coverage across pelagic depth realms.

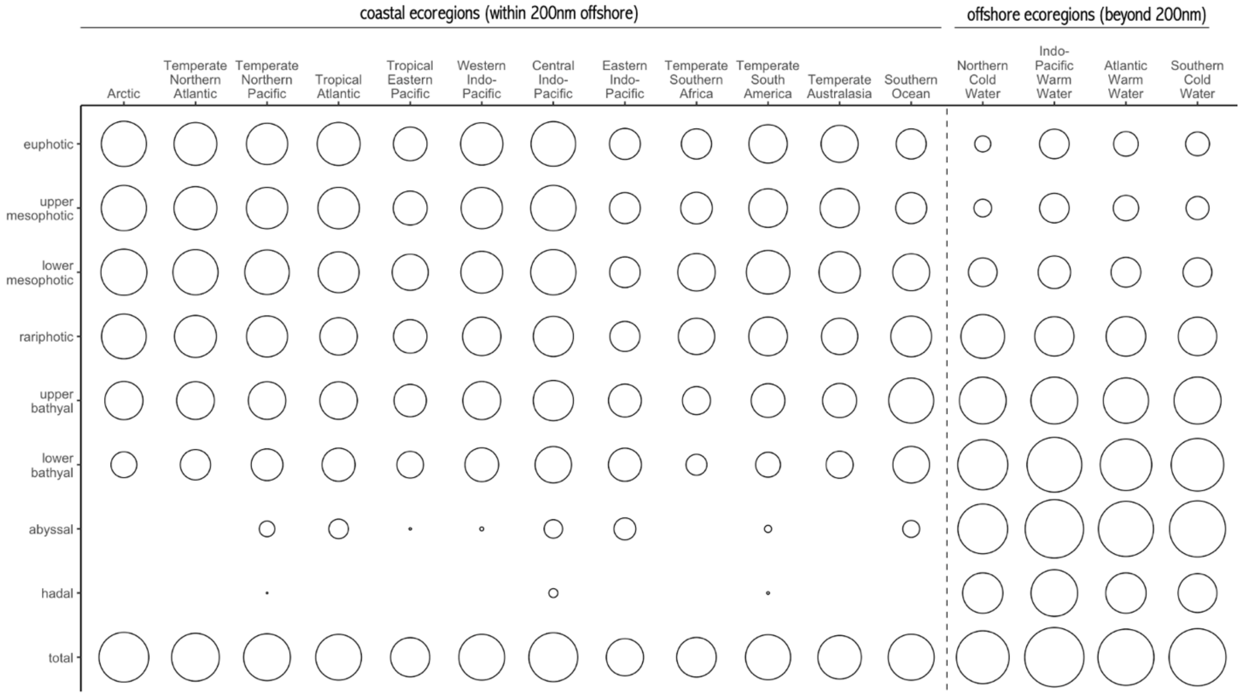

Fig. S4. Relative extent of each 3D realm. The size of circles is proportional to the spatial extent (log km^2) of each 3D realm.

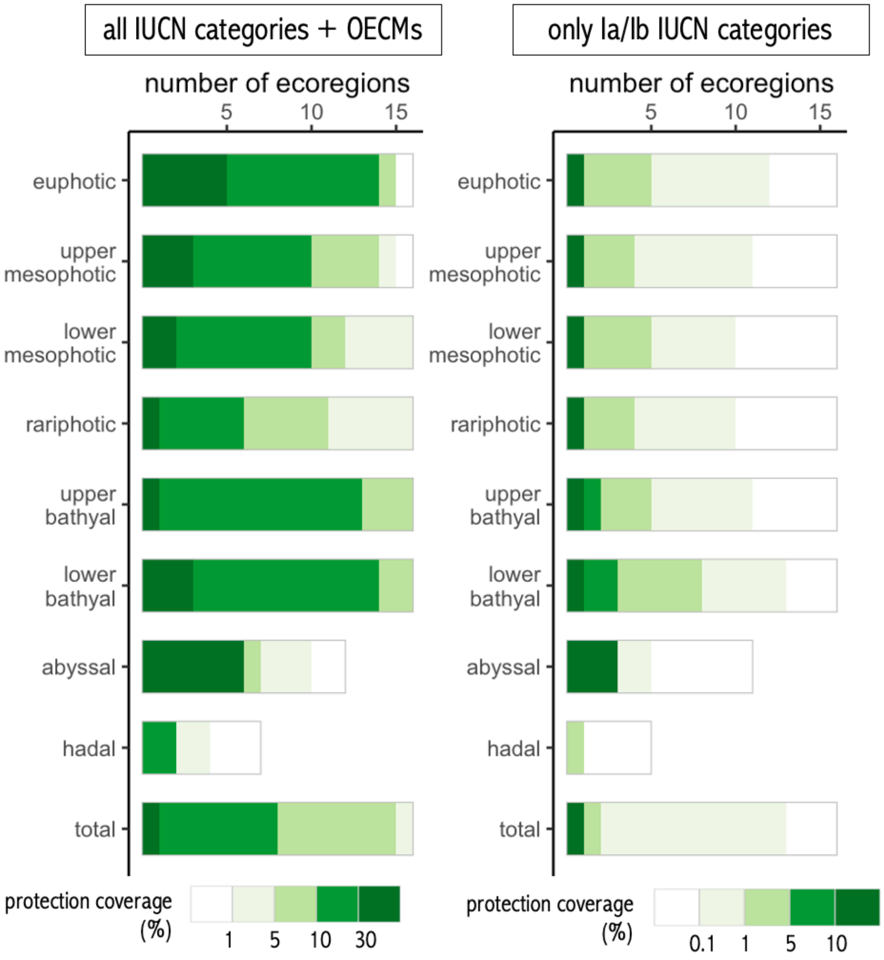

Fig. S5. Number of ecoregions having reached protection coverage targets for each depth realms. Left panel indicates protection coverage across all IUCN categories and OECMs, the right panel indicated protection coverage for Ia and Ib IUCN categories only.

Table S1. Description of IUCN categories as given by the World Database of Protected Areas.

IUCN category	Description
Ia – Strict Nature Reserve	Human visitation, use and impact are strictly controlled and limited to ensure protection of conservation values
Ib – Wilderness Area	Area with minimal human influence protected and managed to preserve their natural condition.
II- National Park	Large natural area managed to protect large-scale ecological processes while allowing for cultural, scientific and visiting activities.
III – Natural Monument or Feature	Area managed to protect a specific natural monument.
IV- Habitat/Species Management Area	Area managed to protect particular species or habitats, often involving active restoration.
V- Protected Landscape/seascape	Area managed to sustainably preserve culturally important human uses and interactions with the landscape.
VI – Protected area with sustainable use of natural resource	Area managed to conserve ecosystems together with associated cultural values and traditional resource management systems.

Table S2. Description of fishing gear categories as given by the Global Fishing Watch.

Gear category	GFW description
fishing	a combination of vessels of unknown fishing gear
fixed gear	a category that includes potential set longlines, set gillnets and pots and traps
pots and traps	vessel that deploys pots (small, portable traps) or traps to catch fish
set longlines	vessel that fishes by setting longlines anchored to the seafloor. These lines have shorter hooked, typically baited, lines hanging from them
set gillnets	vessel that fishes by setting gillnets anchored to the seafloor
trawlers	trawlers, all types
dredge fishing	vessel that tows a dredge and scrapes up edible bottom dwellers such as scallops or oysters
drifting longlines	drifting longlines
driftnets	
pole and line	vessel from which people fish with pole and line
squid jigger	squid jiggers, mostly large industrial pelagic operating vessels
trollers	vessel that tows multiple fishing lines
tuna purse seines	large purse seines primarily fishing for tuna
purse seines	purse seines, both pelagic and demersal
other purse seines	purse seiners fishing for mackerel, anchovies, etc, often smaller and operating nearer the coast than tuna purse seines.
seiners	vessels using seine nets, including potential purse seine vessels targeting tuna and other species, as well as danish and other seines
other seines	danish seines and other seiners not using purse seines.

Table S3. Depth range of fishing gear types registered in the Global Fishing Watch. Table A presents depth range for benthic fishing gear and Table B for pelagic fishing gear.

**A**	traps	dredge fishing	demersal seines	set gillnets	pots	set longlines	bottom trawlers
euphotic (0-30m)	x	x	x	x	x	x	x
up mesophotic (30-60m)	x	x	x	x	x	x	x
low mesophotic (60-150m)		x	x	x	x	x	x
rariphotic (150-300)			x	x	x	x	x
up bathyal (300-1000m)					x	x	x
low bathyal (1000-3500m)							x
abyssal (3500-6000m)							
hadal (< 6000m)							
min depth	5	6	0	15	10	80	20
max depth	50	200	300	150	1000	270	3000

**B**	trollers	driftnets	pole and line	seines	squid jigger	mid-water trawlers	drifting longlines
**epipelagic** (0-200)	x	x	x	x	x	x	x
**mesopelagic** (200-1500)						x	x
**bathypelagic** (1500-3500)							
**abyssopelagic** (3500-6000)							
**hadopelagic** (< 600 m)							
min depth	0	10	0	0	60	10	25
max depth	50	45	30	300	120	1000	350

Table S4. Total area (km^2) and protection coverage (%) across benthic depth realms.

benthic depth realm	lower bathymetric limit (m)	total area (km^2)	protection coverage (all IUCN categories, %)	protection coverage (Ia/Ib categories only, %)	Total fishing effort (10^6 hours $year^{-1}$)
euphotic	-30	6.4E+06	15.1	1.2	15.3 ± 0.9
upper mesophotic	-60	6.6E+06	10.6	0.9	8.9 ± 0.5
lower mesophotic	-150	1.0E+07	8.4	0.6	10.1 ± 0.4
rariphotic	-300	5.9E+06	8.4	1.0	4.2 ± 0.05
upper bathyal	-1000	1.4E+07	16.3	0.6	5.2 ± 0.3
lower bathyal	-3500	8.0E+07	11.9	0.8	3.5 ± 0.04
abyssal	-6000	2.4E+08	5.8	0.6	8.4 ± 0.2
hadal	-11000	3.7E+06	11.1	3.1	0.2 ± 0.03
